# A Bayesian hierarchical logistic regression model of multiple informant family health histories

**DOI:** 10.1186/s12874-019-0700-5

**Published:** 2019-03-12

**Authors:** Jielu Lin, Melanie F. Myers, Laura M. Koehly, Christopher Steven Marcum

**Affiliations:** 1 Northern Arizona University, Flagstaff, AZ USA; 2 Cincinnati Children’s Hospital, University of Cincinnati, Cincinnati, OH USA; 3 National Institutes of Health, Bethesda, MD USA

**Keywords:** Family health history, Multiple informants, Bayesian statistics, Reconciliation

## Abstract

**Background:**

Family health history (FHH) inherently involves collecting proxy reports of health statuses of related family members. Traditionally, such information has been collected from a single informant. More recently, research has suggested that a multiple informant approach to collecting FHH results in improved individual risk assessments. Likewise, recent work has emphasized the importance of incorporating health-related behaviors into FHH-based risk calculations. Integrating both multiple accounts of FHH with behavioral information on family members represents a significant methodological challenge as such FHH data is hierarchical in nature and arises from potentially error-prone processes.

**Methods:**

In this paper, we introduce a statistical model that addresses these challenges using informative priors for background variation in disease prevalence and the effect of other, potentially correlated, variables while accounting for the nested structure of these data. Our empirical example is drawn from previously published data on families with a history of diabetes.

**Results:**

The results of the comparative model assessment suggest that simply accounting for the structured nature of multiple informant FHH data improves classification accuracy over the baseline and that incorporating family member health-related behavioral information into the model is preferred over alternative specifications.

**Conclusions:**

The proposed modelling framework is a flexible solution to integrate multiple informant FHH for risk prediction purposes.

**Electronic supplementary material:**

The online version of this article (10.1186/s12874-019-0700-5) contains supplementary material, which is available to authorized users.

## Background

Many complex diseases are believed to result from the joint influence of genetic, socio-environmental, and lifestyle risk factors that are clustered within families [1], thereby making family health history (FHH) a powerful predictor of varied health outcomes, such as heart disease [2, 3], type 2 diabetes [4–6], and colorectal cancer [7]. To help identify asymptomatic patients who are at increased risk for disease and require additional surveillance and preventive measures, many risk-assessment tools weigh FHH heavily in their algorithms [8–12]. Quantitative risk scores based on an individual’s FHH could even play a deterministic role in diagnosis and treatment decisions [13, 14] and have been the basis for interventions in research [15, 16].

However, much of the enthusiasm of using FHH to enhance preventive screening and care has been dampened by the realization that FHH data, especially those collected from patients’ self-reports, are often incomplete and inaccurate [17–21]. Typically, a patient or research subject reports on their FHH independently and autonomously by informing on the health and disease status of their biological first- and second-degree relatives (e.g., children, siblings, parents, aunts/uncles, and grandparents). This single informant, however, may not have accurate or complete knowledge about their relatives’ disease diagnoses, age at diagnosis, causes of death, and health-related behaviors, leading to an inaccurate risk assessment. This is particularly true for members of the younger generation who have yet to learn about the health of their extended kin [22]. Based on a handful of studies that empirically investigated accuracy of FHH reports [17, 19, 21, 23], sensitivity of self-reported FHH for type 2 diabetes, for example, ranged widely from 53% to 87%, depending on the type and degree of relation between informants and relatives, as well as on methods of external validation (e.g., medical records, interview/questionnaire from relatives).

Recognizing that the predictive value of FHH-based risk scores is likely to be limited by data completeness and accuracy, much effort has focused on improving FHH data collection. One potential remedy for inaccuracies and biases is to focus on the data collection process. It is anticipated that new tools such as pedigree workbooks and online interactive software will encourage individuals to seek their FHH information, thereby improving the accuracy of each individual data point [24, 25].

Since many of these data collection tools employ a shared-model within families, FHH data from multiple members of the same family are available to clinicians and researchers, showing promise in improving risk assessments without having to rely on improving the completeness and accuracy of each individual’s FHH report. Essentially, because FHH reports from related individuals will overlap, such an approach facilitates imputation and cross-validation. It has been shown that incorporating new information from additional sources alone improves risk prediction, yielding an accuracy similar to validating with medical records [26]. While an important step forward, the algorithm used in our previous study is a simple weighted integration of multi-informant FHH without the ability to address uncertainty from individual and/or dyadic characteristics embedded in these data. For example, information provided by each informant may be error-prone and subject to both topical or informant-based uncertainty. Informants may have dissimilar tendencies to make false-positive, false-negative, or missing reports. They may disagree with one another regarding the statuses of the people about whom they are reporting health information. Existing models can address errors and biases arising at the individual level, by including individual attributes as predictors in the regression equation. Such models are not well-suited to dealing with contradicting information at the dyadic level. For example, younger informants tend to have higher rates of missing FHH reports due to generational distance [22]. Women, as kinkeepers, tend to be more knowledgeable about the family’s health information [27–29]. Individuals’ health-related behaviors (e.g., weight, alcohol use) contribute directly to disease risk, and may influence how proactively they seek FHH information [30]. These factors in themselves may not be strong predictors of individual risk, but could signal possible differences in the level of accuracy of FHH data from multiple informants in a family, which in turn can be used to integrate multiple informant FHH (MIFHH) in a meaningful way.

With this type of data, a statistical model used for risk prediction has to not only account for informant-based errors and uncertainty, but also discrepant information provided by different informants by explicitly modeling dependence arising from within- and between-informants. In what follows, we present a statistical model that improves estimation for reconciling discrepant accounts of multiple informant family health histories into a unified FHH that can be used to calculate risk by adjusting for errors arising from the informants, their family members, and background noise. We apply this model to the estimation of individual risk for type 2 diabetes using MIFHH data recently collected from a sample of 45 families residing in the greater Cincinnati area [26]. Specifically, we model the observed diabetes status as dependent on informant-level and dyadic-level attributes and the underlying true diabetes risk as a latent variable that has been observed in two or more informants’ accounts, with informant- and dyadic-level effects.

## Methods

Our goal is to incorporate information from multiple family informants’ family health history observations into a common, integrated FHH. That is, we wish to predict family members’ disease statuses from MIFHH observations and use those estimates to calculate disease risk for unaffected individuals in a family. In the simplest case, we can use arithmetical methods that ignore sources of variation and error in MIFHH data [26]. Alternatively, a statistical model accounting for the process that gives rise to such variation in disease status reports may be used to estimate the integrated FHH. In this section, we introduce a Bayesian hierarchical logistic regression model for improving the precision of such estimation based on MIFHH data.

We begin by defining a common notation. Let each realization of a pedigree containing family health history from *m* informants on *n* family members be represented by **Y**, an *m* × *n*-dimensional matrix. The values of the cells in *Y*_*ij*_ reflect the *i*^*th*^ informant’s report of the *j*^*th*^ family member’s disease status. Ideally, our integration solution reduces the dimensionality of the FHH to a simple *n*-dimensional vector *y* (*y*_1_*,…y*_*n*_).

### Statistical model

We can treat the case of MIFHH integration as a classification problem. Classification models allow the researcher to infer the state of a variable vis-a-vis model parameters and data. We infer one of two states from a set of possibly discrepant observations on a particular individual: does individual *j* truly have a particular disease state (*y* = 1 if yes, and *y* = 0 if no)? Because we do not observe the true disease state on typical FHH data per se, we treat it as a latent variable. Here, we assume informants’ accounts of disease statuses of their family members represent evidence of the underlying true disease state of the individual. While several candidate models for such classification tasks in clinical contexts exist (i.e., Item Response Theory, Naive Bayes, Random Forests), the hierarchically structured and dependent nature of MIFHH data make it particularly challenging to model. Moreover, as disease contexts within families are likely informed by population parameters, better models would incorporate informative priors reflecting this information. As such, we propose using a Bayesian hierarchical logistic regression model that accounts for variability in outcome arising from both informants and the family members they are reporting on, together with informative priors.

Following the Bayesian hierarchical logistic regression models of [31, 32], we assume that individual reports of disease statuses are distributed Bernoulli with probability *θ*. As we have multiple observations from informants on different family members (but not all *m* informants report on all *n* family members), the response vector for each *j*^*th*^ family member is of length *k* × 1, where *k* is the number of informants reporting on *j*, and thus 1 ≤ *k* ≤ *m* ≤ *n*. When the all family members are informants, then *m* = *n*.

Specifically,

1 $$ {y}_{ij}\sim Bern\left({\theta}_{ij}\right),\forall i\in \left(1,2,\dots, k\right) $$

and model *θ*_*ij*_ as a latent variable vis-a-vis the logit-link function (*ϕ* = *ln* $\frac{\mu }{1-\mu }$, where *μ* is the predicted mean vector of the Bernoulli parameter *θ*):

2 $$ \phi \left({\theta}_{ij}\right)={\beta}_0+{X}_{ij}\beta +{W}_i{b}_i+{\epsilon}_{ij}. $$

The first term on the right hand side of Eq. 2 reflects the level-1 intercept (*β*_0_) and the next two terms reflect the matrix of level-1 covariates in *X* and the matrix of level-2 *W* covariates, respectively. These matrices have dimensionality *k* × *p* (*p* being the number of level-1 covariates) and *k* × *q* (*q* being the number of level-2 covariates), respectively. The third term (*ϵ*) captures the errors, which are optionally assumed to be over-dispersed following a normal distribution:

3 $$ {\epsilon}_j\sim N\left(0,{\sigma}^2{I}_{ij}\right), $$

where each *I*_*ij*_ is indexed on identity matrix **I**. In practice, however, the over-dispersion of the errors can be fixed to be 1.

The level-2 effects are also assumed to be distributed normally with mean 0 and covariance *D*:

4 $$ {b}_i\sim {N}_q\left(0,D\right). $$

The conjugate priors for this model as derived by [32] assume that each level-1 effect *β* is distributed normally,

5 $$ \beta \sim {N}_p\left(b,{B}^{-1}\right), $$

where *b* is the mean vector and *B*^− 1^ is the variance of *β*, which can optionally be modeled as Inverse-Wishart if level-1 effects are assumed to be correlated but is here set to be non-informative. Next, the residual error variance follows an Inverse-Gamma distribution,

6 $$ {\sigma}^2\sim IG\left(v,1/\delta \right), $$

where *ν* and *δ* are the shape and scale hyperparameters of the Inverse-Gamma distribution. Finally, we assume that the level-2 effects have an Inverse-Wishart precision matrix prior:

7 $$ D\sim IW\left(\psi, \rho \right), $$

where the scale and shape hyperparameters of the Inverse-Wishart are defined such that *ψ* is a *q* × *q* positive definitive matrix and *ρ* is a scalar such that *ρ* ≥ *q*, respectively. A Kruschke-style diagram of this hierarchical model [33] is depicted in Additional file 1.

Information can be incorporated into these priors by specifying appropriate hyperparameter values. For instance, one may incorporate prior information about the population prevalence of a disease by setting the hyperparameter for the intercept equal to the logit transformed parameter, which would have the result of mixing the observed average reported disease rates in the data with the prior and incorporating that information into the estimate of the model intercept. We sample parameters directly from the posterior of this model using Markov Chain Monte Carlo (MCMC) with the MCMCpack package for R as detailed in [32, 34], which implements Algorithm 2 from [35]. For each model we draw a sample of size 20,000 with a 5000 run burn-in, and sample every other draw with an adaptive mean acceptance rate of about 45%. Thus, our final sample represents 10,000 draws from the posterior of each set of model parameters.

The model described above draws from the posterior of the parameters associated with the informant-informee dyad reports of disease statuses (i.e., at the dyad level). To approximate the equivalent of the individual level reports, we simply average over each individual family member’s vector of posterior predictives (*θ*) as described below.

### Empirical example

The data we use to illustrate our model include MIFHH information collected in 2011–2013 from 128 informants from 45 families residing in the greater Cincinnati area. The number of informants per family ranges from 2 to 5, with an average of 2.8. Each informant independently provided family history of type 2 diabetes for their first- and second-degree biological relatives and we also record self-reports of disease status from the informants. Additionally, each informant provided demographic and lifestyle information such as tobacco and alcohol use and weight status, about each biological relative and themselves. Details about design and data features of this study can be found elsewhere [26]. The final analytic dataset consists of 2159 FHH records contributed by informants from all 45 families, almost two-thirds of which (*n* = 1337) are multiple accounts from informants of the same family with respect to common relatives.

The analysis proceeds in two stages. First, for each family member enumerated we estimate diabetes status as a latent variable with multiple observations provided by different informants using the procedure detailed below. The number of informant based observations per individual family member ranges from 1 to 5. In this model we are able to systematically account for a) population-level prior prevalence of type 2 diabetes, b) family member characteristics (at level-1), and c) informant or family-level variability (at level-2). We assume that the hyperparameters for the mean and variance of *β*_0_ (the level-1 intercept) are ≂ -1*.*99 and 100, respectively. This specification models the population-level prior prevalence of type 2 diabetes by a normal distribution with a mean equivalent to just above observed background probability in the United States (which is roughly 12%, thus − 1*.*99 ≂ *ln* $\frac{0.12}{1-.12}$) and a wide, but finite, variance. We additionally assume vague level-2 covariance priors (with hyperparameters set to *ρ* = *q* and *ψ* = **I** × *q*, which assumes within-informant covariance in reports on family members for the informant level-2 models and within/between informant covariance in the family level-2 models. Finally, for the residual error variances (*σ*^2^), we assume hyperparameters that result in non-informative priors.

Second, we make use of the posterior predictions (*θ*_*ij*_, above) of the final model. These represent the distribution of marginalized model-adjusted probabilities that diabetes status is indicated on the informant-family member dyad. Following [26], we average these predictions over the number of dyads each family member was reported upon by an informant to obtain a weighted estimate of diabetes status.

### Model selection

The primary measure used to compare and select competing parameterizations of our proposed model is the Deviance Information Criteria (DIC). This measure is appropriate as it incorporates a first approximation to the predictive accuracy of the model vis-a-vis the posterior deviance while simultaneously discounting for model complexity. We follow the DIC specification of [31] (pp. 180–3), which defines the DIC as the sum of the average deviance of the posterior sample and one half its variance. The latter term is proportional to the effective number of parameters in the model and is a good estimate of Bayesian model complexity. Like other deviance and likelihood based model selection measures (AIC, BIC, AICC, etc), models with comparatively lower values of DIC are preferred.

We also evaluate classification accuracy for each of our candidate models using the area under the receiver-operator curve (AUC) for both dyadic and individual-level predictions. Larger values of AUC represent better classification, with clinically relevant values exceeding 70% [36]. Additional model robustness checks are reported in Additional file 2.

## Results

Table 1 reports the model fit and predictive power of five candidate models (dyadic data, not aggregated a posteriori to the individual-level). Results for models with two different level-2 covariance structures are reported: one modeling within and between informant covariance (called family level-2) and one modeling within informant covariance (called informant level-2). The null model is effectively an intercept-only logistic regression model with no hierarchical structure and is a natural baseline model by which to compare our candidate models. No AUC is reported for this model as it is degenerate (predictions do not exceed chance under this model). The hierarchical Bayesian logistic regression baseline model (model 1) incorporated only intercept terms for level 1 (dyadic level) and level 2 (informant level). Across all models, the family level-2 was preferred by DIC due to having fewer model parameters and less complexity than the informant level-2 specifications. By contrast, however, the informant level-2 models all exhibit better classification with higher AUCs than the family level-2 models.

**Table 1:** Model fit and classification accuracy of five candidate models from the Hierarchical Bayesian Logistic Regression of MIFHH of Type 2 Diabetes

Terms	Family Level-2			Informant Level-2		
	Pars	DIC	AUC	Pars	DIC	AUC
Null model	3	2508.33	–	3	2508.33	–
Level-1 and Level-2 Intercepts Only	47	2411.2	0.636	130	2465.03	0.694
M1 + Degree of relation^a^ + Same gender^a^	49	2388.91	0.671	132	2457.72	0.708
M2 + Informant gender^b^ + Informant is obese^b^	147	2394.01	0.686	396	2400.52	0.714
M2 + Smokes^a^ + Uses alcohol^a^ + Healthy weight^a^	52	2227.54	0.747	135	2263.68	0.77
M3 + M4	150	2224.39	0.762	399	2237.7	0.78

Note: ^a^dyadic (level 1) attribute; ^b^informant (level 2) attribute

Simply accounting for level-2 heterogeneity improves model fit over the null and results in moderate classification accuracy with an AUC of about 63% for family-level and about 69% for informant-level. In model 2, we add generational distance between the informant and the family member being informed on as well as gender homophily (1 = same gender, 0 = otherwise) to level 1 and observe a slight improvement in both DIC and AUC over model 1. In model 3, we add the informant’s gender (1 = female) and informant’s obesity status (1 = obese, BMI *>* 25) to level 2. This model improves slightly over the previous models by DIC and yet is about as good as a classifier as model 2, with an AUC of about 70% in both cases. Model 3 is also a relatively complex model with a larger number of parameters. In model 4 we add informant’s perspective on the family member’s health-related behavioral risk factors to model 2. This model yielded an improved fit by DIC and had a very good classification accuracy with an AUC around 0.75% for family level-2 and 77% for informant level-2 specifications. Finally, in model 5, we combine the terms from models 3 and 4, which slightly improves DIC by decreases of 3.147 and 25.981, for family and informant level-2 models, respectively. The AUCs for model 5 also improve to about 76% for family level-2 and 78% for informant level-2 models. Despite being a relatively complex model, we prefer model 5 with the informant level-2 covariance structure for the balance of our analyses as it has the best classification accuracy of all models.

**Fig. 1:**
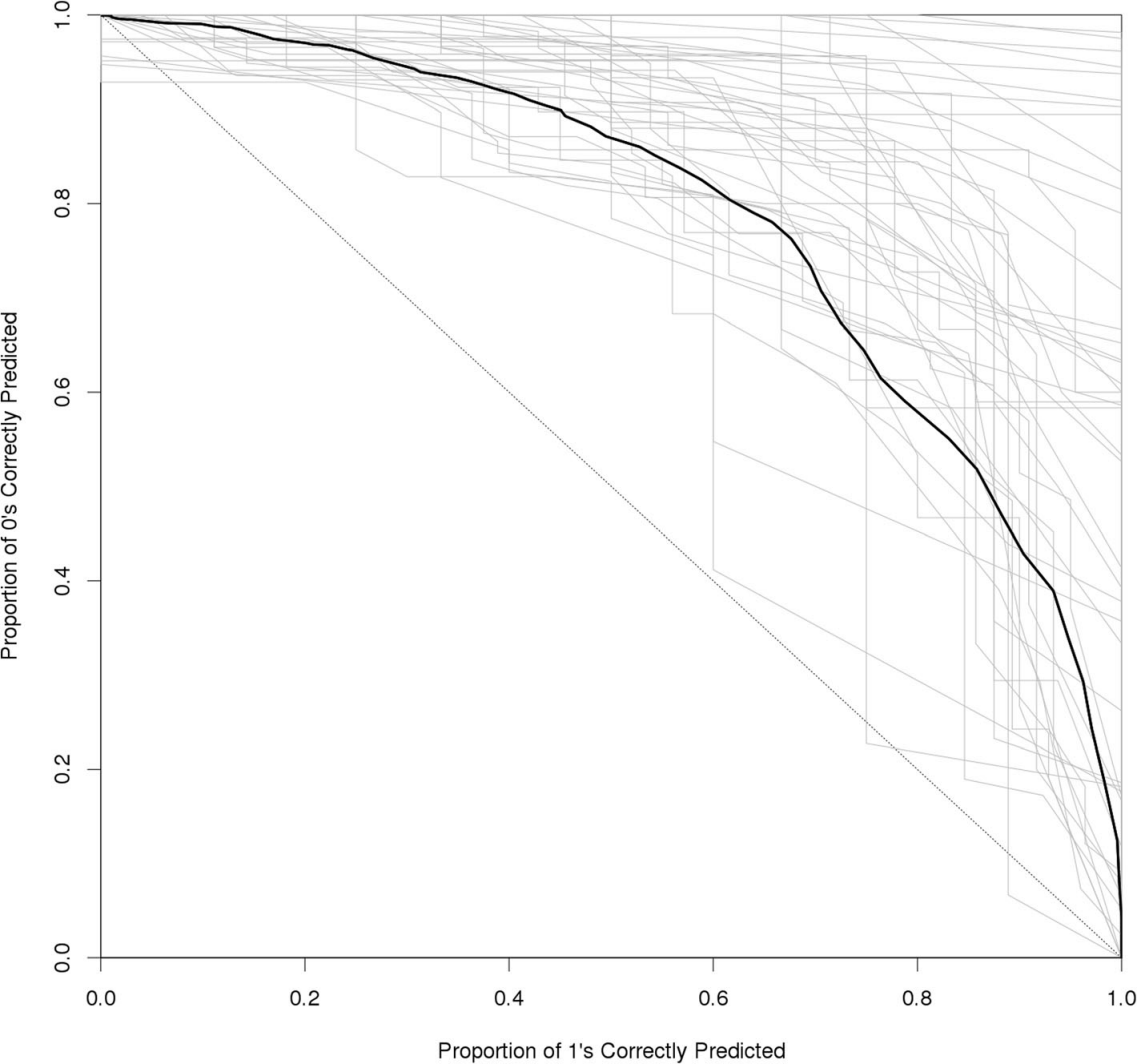
Receiver-Operator Curves (ROC) for Type 2 Diabetes Dyadic Classification from informant level-2 model 5. The thick black line represents the ROC for a model fit with the entire dataset and the thin gray lines represent individual ROCs for each family fit separately

Figure 1 plots the receiver-operator curves (ROCs) for model 5 with the informant level-2. To illustrate the family-level variability around the model fit with the full dataset (indicated by the thick black line) we also stratified the dataset by family and plot separate ROCs for each family model (indicated by the thin gray lines). As the figure demonstrates, model 5 is a good fit in nearly every family separately as well as in the full pooled dataset.

**Fig. 2:**
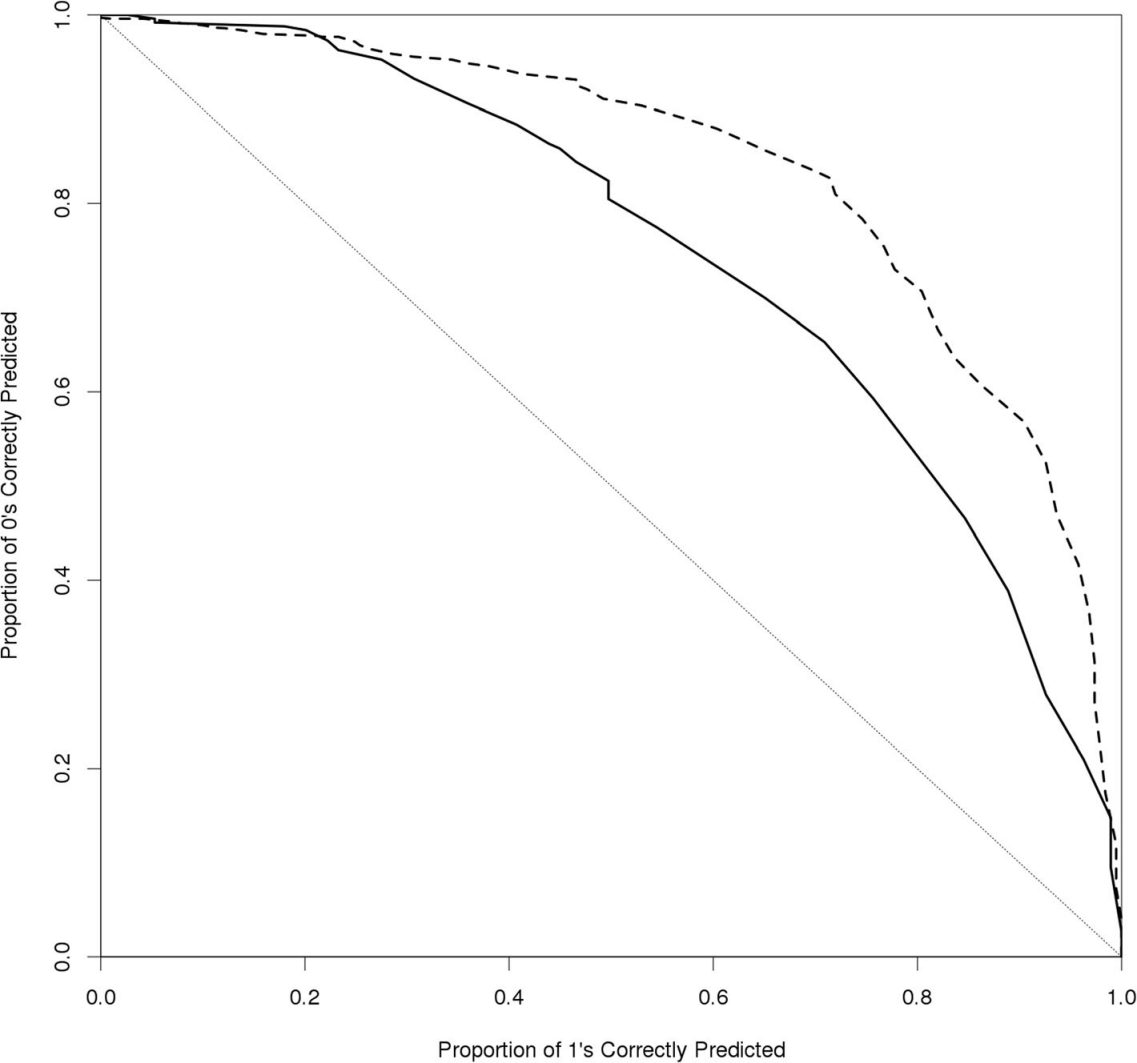
Receiver-Operator Curves (ROC) for Individual Classification. The solid line represents informant level-2 model 1 and the dotted line represents the ROC from model 5, averaging across all informants. These curves represent an AUC of 0.724 for model 1 and an AUC of 0.829 for model 5

**Fig. 3:**
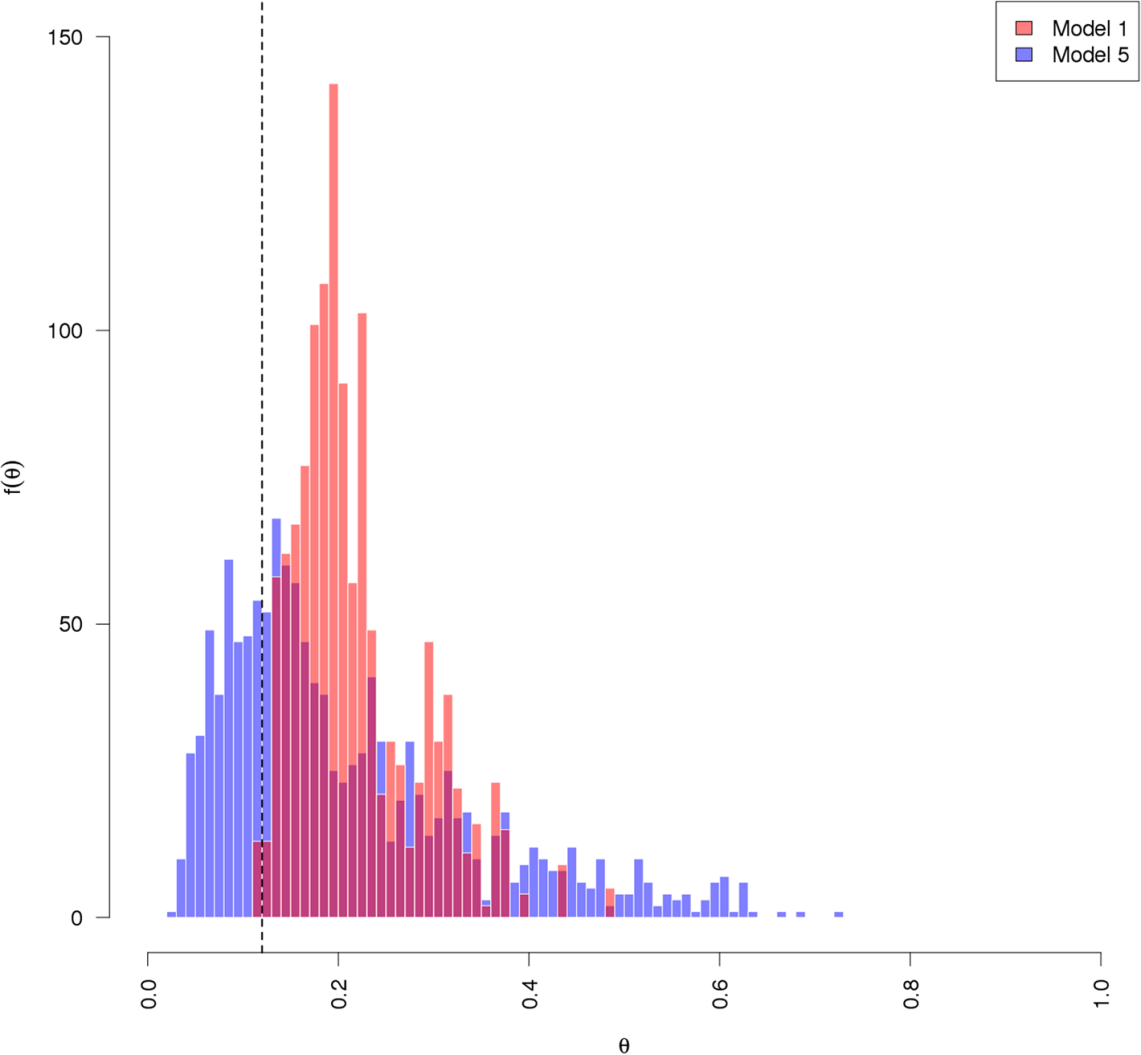
Posterior Predictive Distributions for Models 1 and 5 by averaging the set of each family member’s marginalized latent variables (*θ*) across all informants

After marginalizing over the data and averaging to the individual family member level, we observe very good classification accuracy. For instance, Fig. 2 is a comparison of the ROCs after averaging to the individual level for model 1 (solid line, *AUC* = 0*.*724) and model 5 (dotted line, *AUC* = 0*.*829), which demonstrates the superior classification accuracy of our final model predicting diabetes status for individual family members as a function of background prevalence, multiple informant accounts, and dyadic covariates. Fig. 3 recapitulates these results in terms of the posterior predictive values (i.e., individual predicted probabilities averaged across all informants under the model). The light red histogram represents the posterior predictive values marginalized over the data from model 1 and the light blue histogram represents such from model 5. The vertical dotted line represents the informative prior mean hyperparameter (here *P* (0*.*12)) used to model the background prevalence of diabetes in the population. While model 1, consisting of parameters for only level-1 (family member) and level-2 (informant) intercepts, pools probability mass around the mean of *y*_*ij*_ in the data (*x* ≂ 0*.*22), model 5 which includes covariates, is centered closer to the population prevalence and is more diffuse across the parameter space. In other words, the improvement in the classification accuracy of model 5 over model 1 appears to be the result of its greater representation of heterogeneity in the data.

## Discussion

Increasingly, researchers acknowledge the clustered nature of FHH data and have taken the first steps addressing the problem of FHH data reconciliation using arithmetical methods [26]. While an arithmetical method would be a convenient, straightforward metric for integrating MIFHH into a single FHH, this type of method lacks the ability to incorporate sources of error. In this paper we have described a statistical model for integrating MIFHH into a unified FHH that can be used to calculate individual disease risk scores. The Bayesian hierarchical logistic regression model that we proposed has the advantage of integrating FHH from multiple informants in a more meaningful way, accounting for the processes that gives rise to reporting error and bias in typical FHH data.

Our results reveal two important insights about the nature of FHH data, in general. First, simply accounting for the hierarchical structure of these clustered data in the absence of any covariates improves classification accuracy over the null model (e.g., an improvement from 50 to 70%). This suggests models of FHH are better specified when clustering of family members is incorporated into their estimation. Given that informant error is inherent in any FHH assessment, our findings imply that accounting for such error is an important first step in any FHH-based risk assessment. As such, a latent class approach to estimating the disease status of family members that incorporates clustering of family members into estimation is, at a minimum, necessary for optimal risk evaluation. This is true, whether using FHH information obtained from a single informant or multiple informants.

Second, our best fitting model included informant’s perceptions of the informee’s health-related behaviors. Many clinical and research protocols include information about the informant’s own health-related behaviors (especially, whether they smoke, drink alcohol to excess, and maintain a healthy diet). Our results suggest that collecting information on individuals’ perceptions of their family members’ health-related behaviors may be at least as important in contributing sources of variation in their shared FHH. These covariates may be capturing the joint effect of shared family environment, which are often concealed in a standard disease-centric FHH assessment. Our results suggest that directly accounting for contributions to risk that stem from lifestyle factors as well as heredity yields significant improvement in model fit. Clearly, these proxy reports of health-related behaviors cannot be ignored in current and future models of FHH.

Under the current framework, the model is highly sensitive to the quality of informants reports. Based on current data, our model showed significant improvement in classification accuracy by incorporating multiple informants reports. It is important to point out that this improvement is likely because of high level of agreement between informants. Over 60% of the dyadic comparisons of informants reports are congruent with each other. In this case, a model based on multi-informant information has increased power. Conversely, when inter-informant agreement with respect to a common family members disease diagnosis is low, incorporating multiple reports could lead to more noise (i.e., additional informants reporting “dont know”), and error and bias (i.e., additional informants reporting contradictory information), since the current model weighs each source of information equally.

Both the model and the empirical data have some limitations. First, the approach we’ve taken relies on multiple accounts of FHH from either a single family or from many families. While multiply sourced information is increasingly recognized as important to improving classical approaches to individual risk assessments, these data are difficult to collect and protocols for doing so are outside the current clinical paradigm. In the meantime, our model is probably better suited for research than for practice. The model is also limited in that researchers must choose how the level-2 covariance structure models dependence in families. For the current application, we reported two such sources of dependence: one with just within-informant covariation by clustering at the informant-level and another that incorporated between-informant sources of covariation by clustering at the family-level. While the less complex family level-2 clustered models were preferred by DIC, the informant level-2 models were uniformly better classifiers. In part, this is because the average number of informants per family in our example dataset was small. Increasing the average number of informants will necessitate a more nuanced approach, perhaps one that assumes separate levels of both within- and between-informant covariance in one model. The problem of choosing an appropriate covariance structure for between-family member dependence is non-trivial and future research is needed to evaluate viable alternative specifications. We considered several such specifications in robustness checking but none performed better than the within-informant case we presented here from a classification perspective. Finally, as the sample was drawn from a population that is at increased risk for diabetes, these data and the model evaluation may be an over-characterization of risk profiles in the general population. While we attempted to adjust for this limitation in generalizability with informative priors for the background rate of diabetes, there may be additional sources of unobserved heterogeneity that we cannot account for that make this sample systematically different from the population at large.

Notwithstanding these limitations, the problem we have detailed here is more general than that of the case of MIFHH. The value of such a model lies in enabling users to optimally store, present, and analyze heterogeneous and dynamic FHH data in a way that properly supports clinical risk assessments and treatment decisions. Besides accounts from multiple informants, FHH data can come from different sources, ranging from self-reports, to proxy reports, medical records, or even genomic data. Indeed, a few studies have attempted to further improve FHH-based risk assessment by including molecular genetic variables with promising results [37, 38]. Previously, a similar approach has been used to examine within-patient and between-sample tumor classification accuracy [39]. That model differed from ours, however, in that multiple-modes of tests represented the level-2 source of variation rather than multiple informants. As well, it also lacked the incorporation of covariates. Moreover, information from each of these sources is not fixed in time. New diagnoses, births/deaths, and corrections in family and individual level data often arise and are reported in new accounts of FHH. As efforts to build a core family health history dataset continue [40], there is an urgent need to design platforms with the capacity to reconcile and integrate FHH from multiple sources in a dynamic manner. The model we’ve proposed here is one example of how such a database may be leveraged for risk prediction in future work.

## Conclusion

The proposed modelling framework is a flexible solution to integrate multiple informant FHH for risk prediction purposes. Our approach contributes to the state of the science on model-based risk assessments by allowing for the joint incorporation of various forms of correlation structure within families, together with population-level priors, and individual attributes. This framework allows to more fully capture the context of how multiple FHH reports shape disease risk assessments over existing methods. Our empirical example results indicate that, for type-2 diabetes, both disease history and health behavior information should be collected for more accurate clinical and research assessments of FHH.

## Additional files

Additional file 1: Model diagram. (DOCX 125 kb)

Additional file 2: Supplementary analysis for model robustness. (DOCX 43 kb)

## Abbreviations

AUC: Area Under the Curve
BMI: Body Mass Index
DIC: Deviance Information Criteria
FHH: Family Health History
MCMC: Markov Chain Monte Carlo
MIFHH: Multiple Informant Family Health History
ROC: Receiver Operator Curve
