# Supplementary material for: A Bayesian hierarchical logistic regression model of multiple informant family health histories
Source: BMC Med Res Methodol. 2019 Mar 12;19:56. doi: 10.1186/s12874-019-0700-5 (PMC6419428; doi:10.1186/s12874-019-0700-5)
Supplement: Supplementary file 1 — Model diagram. (DOCX 125 kb) [file 12874_2019_700_MOESM1_ESM.docx]

### Appendix A: Model diagram

Figure [4](#x1-210014) provides a graphical depiction of the Bayesian Hierarchical Regression Model in the style of [[33](#Xkruschke2015bayesian)] as specified in the empirical example with informative hyperparameters on the prior for β_0_ and non-informative priors otherwise. The prior for the covariance matrix of level-1 effects, B^-1^, is also distributed inverse-Wishart (not shown here as it is set to be non-informative).


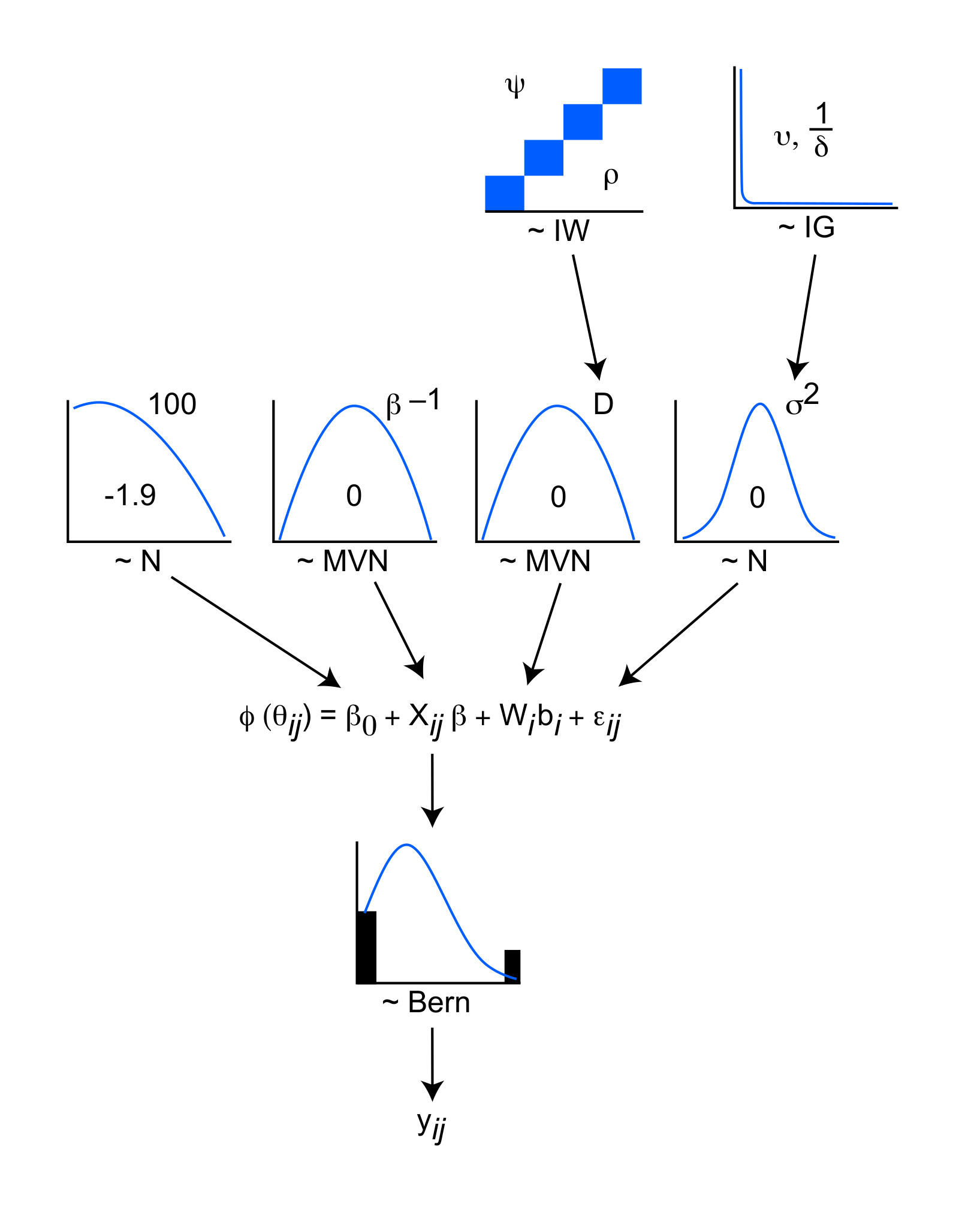


Figure 4: Kruschke-Style Diagram of Proposed Model
