# Supplementary material for: A Bayesian hierarchical logistic regression model of multiple informant family health histories
Source: BMC Med Res Methodol. 2019 Mar 12;19:56. doi: 10.1186/s12874-019-0700-5 (PMC6419428; doi:10.1186/s12874-019-0700-5)
Supplement: Supplementary file 2 — Supplementary analysis for model robustness. (DOCX 43 kb) [file 12874_2019_700_MOESM2_ESM.docx]

### Appendix B: Supplementary analysis for model robustness

To assess the robustness of the model as a classifier, we iteratively divided our data into training and testing subsets by uniformly downsampling observations from 10% to 50% (in increments of 10%) of the original dataset. We then estimated the model 5 on each resulting training set and marginalized the testing set observations over the training model parameters to generate posterior predictions of the testing set observations given the training model. AUCs from the receiver operator curve of each trial were then calculated. This process was repeated 500 times. Box-and-whisker plots of the resulting differences in AUCs between the training and testing sets are reported in Figure [5](#x1-220015), grouped by downsampling rate. The figure demonstrates that the model performs reasonably well as a classifier in the face of new data. On average, the testing set resulted in AUCs of about 0.71 (min=0.59,max=0.83), which was about 10% lower than the average training set AUCs of 0.78 (min=0.72, max=0.83). On average, 97% of the tests resulted in training set AUCs being greater than the testing set AUCs while the mean difference between them was about 0.07. Thus, while, as expected, the AUCs of the training set were not as well classified as the testing set across all sample rates, the difference between them were relatively small on average. This suggests that the model is relatively robust to new data as a classifier.


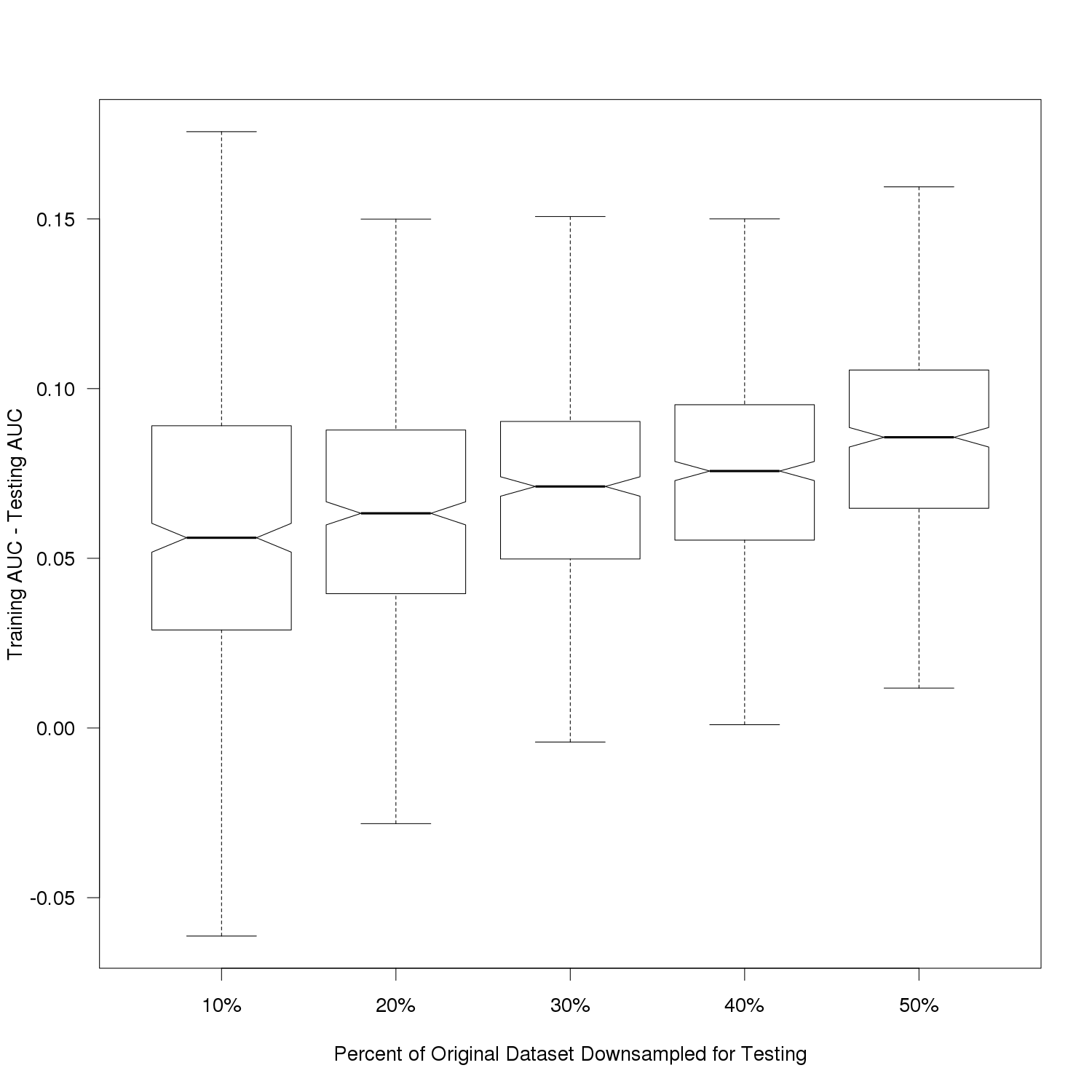


Figure 5: Box-and-Whisker Plots of the Distribution of Differences Between Testing and Training Areas Under the Receiver Operator Curves
